# “Mesenchymal stem cells”: fact or fiction, and implications in their therapeutic use

**DOI:** 10.12688/f1000research.10955.1

**Published:** 2017-04-20

**Authors:** Pamela Robey

**Affiliations:** 1 Skeletal Biology Section, National Institute of Dental and Craniofacial Research, National Institutes of Health, Department of Health and Human Services, Bethesda, MD, 20892, USA

**Keywords:** mesenchymal stem cell, MSC, tissue-specific stem/progenitor cells, tissue regeneration, tissue engineering

## Abstract

The concept of a post-natal “mesenchymal stem cell” (“MSC”) originated from studies focused on bone marrow stromal cells (BMSCs), which are non-hematopoietic adherent cells, a subset of which are skeletal stem cells (SSCs), able to form cartilage, bone, hematopoiesis-supportive stroma, and marrow adipocytes based on rigorous clonal and differentiation assays. Subsequently, it was speculated that BMSCs could form other mesodermal derivatives and even cell types from other germ layers. Based on BMSC surface markers, representative of fibroblastic cells, and imprecise differentiation assays, it was further imagined that “MSCs” are ubiquitous and equipotent. However, “MSCs” do not have a common embryonic origin and are not a lineage, but recent studies indicate that they are tissue-specific stem/progenitor cells. These cells share cell surface features owing to their fibroblastic nature, but they are not identical. They display different differentiation capacities based on their tissue origin but do not “trans-differentiate” outside of their lineage, based on rigorous assays. For these reasons, the “MSC” term should be abandoned. Tissue-specific stem/progenitor cells provide the opportunity to devise methods for tissue regeneration by the cells themselves (tissue engineering). Their use in other forms of regenerative medicine based on paracrine, immunosuppressive, and immunomodulatory effects is far less clear.

## Introduction

One of the best examples (and perhaps the first) of the existence of a post-natal stem cell within a connective tissue comes from the pioneering work of Alexander Friedenstein, later working with Maureen Owen. They found that when single-cell suspensions of bone marrow are plated at clonal density, there is a rapidly adherent fibroblastic cell originating from bone marrow stroma that can establish a colony in a density-independent manner. When the progeny (bone marrow stromal cells [BMSCs]) of these colony-forming unit fibroblasts (CFU-Fs) were transplanted *in vivo* in a diffusion chamber (a closed system), cartilage formed in the relatively anaerobic interior and bone on the relatively aerobic exterior of the diffusion chamber. When transplanted *in vivo* in conjunction with an appropriate scaffold (an open system), the colonies recreated a bone/marrow organ composed of bone, osteocytes, osteoblasts, hematopoiesis-supportive stroma and marrow adipocytes of donor origin, and hematopoiesis of recipient origin (reviewed in 1). Based on these assays (indicating multipotency of the progeny of CFU-Fs by rigorous differentiation assays) and others that followed who demonstrated self-renewal, it is clear that bone marrow stroma contains a bona fide skeletal stem cell (SSC) capable of reforming skeletal tissues ^2, 3^.

## A change in terminology and concept

The initial (and rigorous) concept of a tissue-specific SSC/BMSC population was subsequently modified to suggest, without experimental evidence, that SSCs/BMSCs could form other mesodermal tissues such as muscle, tendon, ligament, etc., by a “mesengenic process”, and the cells were subsequently renamed “mesenchymal stem cells” (“MSCs”) ^4^. However, “mesenchyme” is primarily a histological term to describe a transient embryonic connective tissue arising primarily from mesoderm but also from neural crest of ectodermal origin. Consequently, “mesenchyme” is not synonymous with “mesoderm”, and the terms cannot be used interchangeably. Furthermore, embryonic mesodermal mesenchyme develops not only into connective tissues but also into blood and blood vessels ^5^. There is no post-natal stem cell that has this ability based on rigorous assays. Using bone as an example, there are at least three different sources of bone during embryonic development: neural crest (facial bones), paraxial mesoderm (axial bones), and somatic lateral plate mesoderm (appendicular bones) (reviewed in 6). Thus, there is no single embryonic origin for bone, so how could it be that there is a common “MSC” for all connective tissues?

## Use and abuse of BMSC surface markers and differentiation assays

In spite of these incongruities, bone marrow-derived “MSCs” became a point of interest for many, based on the “mesengenic” process, and a vast number of studies identified a variety of cell surface markers that are expressed by BMSCs in hopes of developing methods to more efficiently isolate them. These cells are uniformly negative for hematopoietic and certain endothelial cell markers and are positive for a long list of others (reviewed in 7, 8). However, these markers are not specific, either individually or in combination. They are expressed by many adherent fibroblastic cells, even those that are not stem cells based on clonal analysis and rigorous differentiation assays. Furthermore, the level of expression of many of these markers changes with time in culture and, consequently, their use is best confined to freshly isolated cells rather than those that have been *ex vivo* expanded ^9, 10^.

Because of the lack of specificity of these markers, a plethora of studies emerged suggesting that “MSCs” can be isolated from virtually any tissue ^11^. These studies were further confounded by the use of *in vitro* assays that suggested that “MSCs” from non-skeletal tissues are capable of forming cartilage, bone, and fat. However, these assays are rarely applied to clonal populations of cells and are highly prone to artifact or misinterpretation. For the osteogenesis assay, alizarin red S cannot distinguish between dystrophic calcification induced by dead and dying cells versus matrix mineralization. Furthermore, if the cells make the enzyme alkaline phosphatase, it cleaves β-glycerophosphate, a component of osteogenic differentiation medium. When the phosphate concentration in the medium becomes high enough, calcium phosphate precipitates, and it too stains with alizarin red S, but it is not hydroxyapatite ^12, 13^. In addition, many studies treat cells with bone morphogenetic proteins (BMPs) or genetically modify them to force the expression of osteogenic transcription factors. However, BMPs will induce an (often temporary) osteogenic phenotype in any fibroblastic cell, as has been known from the pioneering work of Marshall Urist ^14^ and those who followed. BMP treatment and/or genetic engineering cannot be used as proof that non-skeletal “MSCs” are inherently osteogenic. In the adipogenic assay, many cells take up lipid from the serum in the medium and do not synthesize lipids *de novo* ^15^. *In vivo* transplantation with an appropriate scaffold is the gold standard by which to assess osteogenic and adipogenic differentiation ^13^. For chondrogenesis, the *in vitro* cell pellet culture is the current gold standard, and one must see bona fide chondrocytes lying in lacunae, surrounded by extracellular matrix that stains purple with toluidine blue (metachromasia) ^16, 17^. What many reports show are pellets of dead cells that are barely stained with alcian blue, which will also lightly stain osteoid. Safranin O is also often used because it will stain glycosaminoglycans linked to aggrecan, the cartilage-specific proteoglycan, but it also stains DNA.

In spite of these caveats, the current position of the ISCT lists the minimal criteria for “MSCs”, now renamed “mesenchymal stromal cells”, as follows: 1) plastic-adherent cells in standard culture conditions, 2) expression of CD73, CD90, and CD150 and lack of expression of CD11b, CD14, CD19, CD34, CD45, and HLA-DR molecules, and 3) differentiation into chondrocytes, osteoblasts, and adipocytes *in vitro* ^18^ (note the absence of requirements for clonal analyses and appropriate *in vivo* studies).

## Pericyte origin of SSCs as identified by CD146 expression

In spite of the fact that it is not specific for SSCs/BMSCs, CD146 is emerging as a useful marker for the identification of human SSCs ^3^, although it must be noted that mouse SSCs appear to express different markers. An initial study by Bianco and coworkers revealed that sorting of freshly isolated human bone marrow for CD45 ^–^/CD34 ^–^/CD146 ^+^ efficiently isolates all the CFU-Fs, but not all the colonies generated by the CD146 ^+^ CFU-Fs were multipotent based upon *in vivo* transplantation. Approximately 10% of the single colony-derived strains were able to recreate a bone/marrow organ (multipotent), while the remainder formed only bone or fibrous tissue ^3^. Thus, not even all CFU-Fs are multipotent. However, the *in vivo* identity and localization was still to be determined. Because it is known that CD146 is also expressed by endothelial cells, this study took advantage of a human-specific CD146 antibody to localize human cells in the transplants generated in immunocompromised mice. This antibody identified the human CD146 ^+^ cells as pericytes, cells that wrapped around blood vessels of mouse origin. Human cells re-isolated from these transplants were clonogenic and were again shown to express CD146, providing evidence for self-renewal ^3^. ****The notion that pericytes are tissue-specific stem/progenitor cells in bone marrow and a part of the hematopoietic stem cell niche is further supported by an increasing number of studies using mouse reporter lines such as *Nes*-GFP and *Lepr*-GFP and lineage tracing with *Nes*-Cre ^ER^ and *Lepr-*Cre, just to name a few because of space constraints (see 19 for more information). However, there are a number of issues related to mouse studies that have yet to be fully resolved with regard to the most appropriate marker and study design to use. In addition, there are significant differences between mouse and human SSCs/BMSCs: e.g. CD146 isolates all CFU-Fs from human bone marrow but not from mouse bone marrow ^20^.

A recent study used the identical sorting strategy as in Sacchetti *et al*. (CD45 ^–^/CD34 ^–^/CD146 ^+^ cells, hereafter referred to as CD146 ^+^ cells) to isolate CFU-Fs from non-skeletal tissues (human muscle, cord blood, and others). The cells from these different tissues, grown under identical conditions, were clonogenic and expressed all the “MSC” markers; however, transcriptome analysis revealed that the cells were different: bone marrow-derived CD146 ^+^ cells expressed osteogenic transcription factors, and muscle-derived CD146 ^+^ cells expressed myogenic transcription factors. Rigorous differentiation assays confirmed the commitment of these cells to a specific lineage: bone marrow-derived CD146 ^+^ cells formed a bone/marrow organ upon *in vivo* transplantation but did not spontaneously form myotubes *in vitro*, and muscle-derived CD146 ^+^ cells did not form bone *in vivo* but did spontaneously form myotubes *in vitro* in the absence of exogenous myoblasts. Using an *in vivo* transplantation assay after muscle injury, human muscle-derived CD146 ^+^ cells were also found to self-renew. Interestingly, CD146 was found to localize to pericytes not only in bone marrow but also in muscle. The importance of bone- and muscle-derived CD146 ^+^ cells (GFP-labeled) in the formation and stabilization of blood vessels was verified by co-transplanting them along with human endothelial cells in Matrigel ^TM^ plugs into immunocompromised mice. Examination of these plugs revealed GFP-labeled human CD146 ^+^ cells surrounding human CD34 ^+^ endothelial cells to form capillary-like structures, which successfully joined with blood vessels of mouse origin, as visualized by their engorgement with red blood cells ^21^.

## The developmental process by which pericytes are formed

Taken together, the results from these studies led to the hypothesis that local stem/progenitor cells are pericytes that are formed by what may be a developmental process that is shared by many connective tissues ^6, 9, 21, 22^. During development, blood vessels are initially devoid of pericytes and are unstable ^23^ (reviewed in 24, 25). As blood vessels invade a developing tissue, they capture local cells that have certain cell surface characteristics (i.e. that of fibroblastic cells) that are committed to a lineage and incorporate them as pericytes, giving the blood vessels stability. These captured cells remain quiescent until liberated from the blood vessel wall owing to an injury or in response to the need for tissue turnover, at which point they then reform the cell types of that tissue. Additional studies using CD45 ^–^/CD34 ^–^/CD146 ^+^ derived from other tissues are needed to determine how generalized this process is. However, that is not to say that all pericytes are stem cells, as shown by clonal analysis and rigorous differentiation assays, nor are they a lineage (pericytes from different tissues do not arise from a common embryonic precursor) ^25^, just as “MSCs” are not a lineage. They are tissue-specific stem/progenitor cells, and they do not “trans-differentiate” outside of their lineage without extreme manipulation.

## The “MSC” term should be abandoned

These studies also highlight that while there are assays that can commonly be applied to study tissue-specific stem/progenitor cells (e.g. colony forming efficiency, cell surface analysis, and transcriptome analysis), rigorous differentiation assays must be tailored to the tissue of origin (there is no one assay that can be used for cells derived from all tissues). For example, osteogenic and adipogenic differentiation must be determined by *in vivo* transplantation with suitable scaffolds, whereas, currently, chondrogenic differentiation is best assessed *in vitro*. Likewise, the most rigorous myogenic assay is also *in vitro* and performed in the absence of exogenous myocytes. Myocytes will fuse to form myotubes with many cell types *in vitro* and *in vivo*, but that does not qualify the donor cells as being inherently myogenic ^26^. For all these reasons, it would be appropriate to abandon the term “MSC”. There is no common, ubiquitous, equipotent “MSC” in the post-natal organism. The terminology should be based on their tissue of origin for clarity: e.g. BMSCs (a subset of which are SSCs), adipose-derived stromal cells (ADSCs), umbilical cord blood-borne fibroblasts (UCB-BFs), etc. Most certainly, the distinction between a stromal cell and a fibroblast is nebulous at best, and these terms are often used interchangeably. However, in these particular examples, there is a distinction between BMSCs and ADSCs, which are functionally supportive structures of soft tissues (marrow and adipose, respectively) versus UCB-BFs, which are not. Of note, many of the reported “MSC” populations have not been adequately assessed by clonal analysis to prove the existence of a subset of multipotent (or unipotent) cells that can self-renew (extensive proliferation is not evidence of self-renewal). Some may suggest the use of the term “pericyte” to indicate the location of tissue-specific stem/progenitor cells. Pericytes are generally defined by location, morphology, and the expression of certain genes (although they are not pericyte-specific) and are described as cells with long processes that wrap around endothelial tubes to provide stability ^25^. However, more studies of pericytes from different tissues are needed to confirm potency and the ability of these cells to self-renew, the two defining features of a stem cell, and the name of the population of cells (fibroblasts, stromal cells, or pericytes) should be determined on a case by case basis.

One may ask, why does the name matter? First, it is a matter of rigorous basic biology to recognize the inherent differences between local tissue-specific stem/progenitor cells, with more focus on their role in tissue homeostasis, their role in pathogenetic mechanisms of disease, and their use in tissue engineering whereby the cells themselves recreate their tissue of origin. Second, despite all the issues related to “MSC” biology, they are being used extensively and interchangeably in human clinical trials (>600 clinical trials using “MSCs” listed in clinicaltrials.gov using the search term “mesenchymal stem cell” OR “mesenchymal stromal cell”) for the treatment of spinal cord injuries, multiple sclerosis, Sjögren’s syndrome, nephropathies, amyotrophic lateral sclerosis (ALS), and ocular disorders, as examples, many without a clear rationale. Furthermore, “MSCs” are not swappable, either in the study of tissue-specific homeostasis (“MSCs” from adipose cannot substitute for BMSCs to study mechanisms of bone formation) or in their medical use (“MSCs” from muscle cannot substitute for BMSCs in rebuilding bone).

## Distinguishing between “stem cell” and cell therapies

To date, there are only a few examples of successful bona fide stem cell therapies: blood reconstitution with populations containing hematopoietic stem cells ^27^, corneal regeneration by populations of limbal cells containing limbal stem cells (reviewed in 28), skin regeneration with epidermal stem cells that contain stem cells (reviewed in 29), and a number of small studies regenerating bone with SSCs/BMSCs ^30^. While it is unlikely that “purified” stem cells would be used directly for tissue regeneration owing to their rarity, it is important to document the presence of a stem cell subset, which is required for appropriate tissue turnover ^12^. On the other hand, the notion emerged that SSCs/BMSCs (and other types of “MSCs”) could be infused systemically or locally injected to treat generalized diseases and disorders or injuries. Initially, a long list of studies suggested that these infused cells could “trans-differentiate” into cells outside of their lineage (e.g. SSCs/BMSCs could form neurons, cardiomyocytes, etc.) based on the expression of a few markers. Subsequently, more rigorous studies that followed indicated that trans-differentiation is a rare event, if it occurs at all, and proof of functionality of these trans-differentiated cells was lacking. Yet some studies reported beneficial effects of “MSCs” in treating a long list of diseases and disorders in animal models and in humans. It was hypothesized that infused or directly injected cells exert paracrine effects that encourage local stem/progenitor cells to begin the repair process or that they were exerting immunomodulatory and immunosuppressive effects that would bring about improvement ^31^. However, it is well known that upon systemic infusion, “MSCs” of all types are rapidly cleared by the lungs and rarely escape from the circulation. They rapidly disappear, even upon direct injection without a scaffold or carrier ^32^. Consequently, the mechanism(s) of action have not been well elucidated and are very unclear. Furthermore, these putative effects have not been pinpointed to the rare subset of stem cells that are present within any “MSC” population and cannot be correctly called a “stem” cell therapy. The putative effects are brought about by the entire cell population. In addition, it is also not clear that “MSCs” are unique in this regard, as it has been demonstrated that skin fibroblasts exert similar effects ^33^. Many studies have not used a “negative control” cell type to show the specificity of “MSCs” in these treatments.

Nonetheless, these putative effects prompted another name change to “medicinal signaling cells”, a term meant to portray these cells not as stem cells but as paracrine perivascular cells spread throughout the body that function only following injury and inflammation by secreting factors to dampen the immune system on one side and factors that encourage tissue regeneration on the other side ^31^. This name change and the explanation for it have done little to clarify the field. First, it has been demonstrated that some pericytes are, in fact, stem cells as described above. Second, virtually every cell type in the body exerts paracrine effects (and, to some extent, immunomodulatory and immunosuppressive effects as well). Third, this re-definition does not explain how these cells would work when they do not survive infusion or injection. Lastly, if the putative beneficial effects are based on secreted factors, efforts to identify these factors would eliminate the need for cell infusion or injection, which does have some risk.

## Unauthorized “stem cell” clinics

In addition to the studies listed in clinicaltrials.gov, there are ~600 clinics in the USA alone that are utilizing various “stem” cell populations, including “MSCs” (most commonly the patient’s own adipose- and bone-marrow-derived “MSCs”) for various treatments, many without IRB- and FDA-approved protocols and paid for by the patients themselves ( https://www.scientificamerican.com/article/unproved-stem-cell-clinics-proliferate-in-the-u-s/). Of note, liposuction and bone marrow aspiration are raw samples, containing many types of cells, the vast majority of which are not stem cells. Furthermore, it has been difficult to assess the efficacy of these “therapies” because many of the diseases and disorders that are being treated wax and wane. In addition, there is generally a lack of systematic reporting of outcomes. Nonetheless, it is argued by these clinics and associated companies that these treatments are allowable under current FDA guidelines because the cells are “minimally manipulated”. These arguments, along with the intense desire of patients with various diseases and disorders for the rapid development of new therapies, have resulted in the proposal of new legislation (the REGROW Act, https://www.congress.gov/bill/114th-congress/senate-bill/2689) that would reduce the regulatory burden and allow patients to gain more rapid access to therapies that are still experimental. Concurrently, the FDA was drafting four new guides to clarify certain aspects of the regulations for human cell- and tissue-based products. A public hearing was held on 12–13 September 2016, with a long list of presenters representing patients’ perspectives, along with requests from investigators for clarifications of these guidelines with respect to terminology such as structural versus non-structural use, homologous use, and a plea for the alternative regulation of autologous cell products with the use of adipose-derived “stem” cells heading the list ( https://videocast.nih.gov/summary.asp?Live=19816&bhcp=1). In an article published in NEJM ^34^, the FDA reiterated that there is indeed a great deal of excitement for the development of stem cell therapies but noted that there have been a number of serious adverse events associated with unproven treatments, including some with “MSCs” being used with IRB approval, highlighting the need to rigorously define the benefits and risks in order to provide patients with safe and effective treatments. Undoubtedly, much more discussion will ensue as this field moves forward.

In summary, it is now beginning to be recognized that several post-natal tissues contain “tissue-specific stem/progenitor cells” (not to be called “MSCs” in any of its iterations). This term is factual: it eliminates reference to an embryonic tissue, it makes no erroneous assumptions on their “commonality” or “ubiquitous” nature, and it highlights their cell character and, most importantly, their restricted differentiation capacity. Tissue-specific stem/progenitor cells are pericytes in a number of tissues that arise during what might be a common developmental process, although more tissues need to be examined. Tissue-specific stem/progenitor cells have the ability to recreate the tissue from which they are isolated and represent a valuable tool for studying tissue dynamics in health and disease and for use in tissue engineering. At this time, the scientific community should open up discussions to establish an appropriate nomenclature for these cells for the sake of clarity, taking into account that there is no biological sense in trying to group disparate populations of cells under a common name, even as a nickname. The use of tissue-specific stem/progenitor cells in other forms of regenerative medicine based on non-stem cell functions (paracrine, immunosuppression, immunomodulation) is an area that will require much more rigorous evaluation for not only safety but also determination of the mechanism(s) of action and efficacy.

## Declaration

The expressed opinions are those of the author and not necessarily those of the National Institutes of Health.
